# Benchtop and Animal Validation of a Projective Imaging System for Potential Use in Intraoperative Surgical Guidance

**DOI:** 10.1371/journal.pone.0157794

**Published:** 2016-07-08

**Authors:** Qi Gan, Dong Wang, Jian Ye, Zeshu Zhang, Xinrui Wang, Chuanzhen Hu, Pengfei Shao, Ronald X. Xu

**Affiliations:** 1 Department of Precision Machinery and Precision Instrumentation, University of Science and Technology of China, Hefei, Anhui, People’s Republic of China; 2 Department of Biomedical Engineering, The Ohio State University, Columbus, Ohio, United States of America; School of Medicine, Fu Jen Catholic University, TAIWAN

## Abstract

We propose a projective navigation system for fluorescence imaging and image display in a natural mode of visual perception. The system consists of an excitation light source, a monochromatic charge coupled device (CCD) camera, a host computer, a projector, a proximity sensor and a Complementary metal–oxide–semiconductor (CMOS) camera. With perspective transformation and calibration, our surgical navigation system is able to achieve an overall imaging speed higher than 60 frames per second, with a latency of 330 ms, a spatial sensitivity better than 0.5 mm in both vertical and horizontal directions, and a projection bias less than 1 mm. The technical feasibility of image-guided surgery is demonstrated in both agar-agar gel phantoms and an ex vivo chicken breast model embedding Indocyanine Green (ICG). The biological utility of the system is demonstrated in vivo in a classic model of ICG hepatic metabolism. Our benchtop, ex vivo and in vivo experiments demonstrate the clinical potential for intraoperative delineation of disease margin and image-guided resection surgery.

## Introduction

For many cancer patients, surgery remains one of the most effective treatment options. Advanced surgical navigation techniques for accurate assessment of tumor resection margin may support important decision making in an operating room (OR). The urgent clinical need for surgical navigation can be exemplified by breast-conserving surgery (BCS). BCS is a common method for treating early stage breast cancer [1, 2]. However, up to 20% to 50% BCS patients require a re-excision procedure, leading to unnecessary patient stress, suboptimal cosmetic outcome, and increased local recurrence rates [3–5]. Since failure to obtain a clean margin contributes to the high re-excision rate, it is important to provide the surgeon with real-time, precise, and cancer-specific information about surgical margin. Histopathologic analysis is the gold standard for cancer margin assessment. However, conventional histopathologic evaluation is time-consuming, costly, and easy to miss the residual lesions due to specimen sampling error. Consequently, patients with pathologically negative surgical resection margins after BCS still have a local recurrence rate ranging from 7% to 27% [6].

In recent years, near-infrared fluorescence (NIRF) imaging techniques have been widely explored for non-invasive and real-time detection of various tissue malignancies, such as ovarian carcinomas [7], sentinel lymph nodes [8–10], hepatocellular carcinomas [11], lymphatic systems [12, 13], and breast lesions [14, 15]. Various NIRF systems are now available for preclinical and clinical studies [16], including those with polarization imaging capabilities [17]. For example, the SPY system (Novadaq Technologies, Toronto, Canada) integrates a camera unit, a laser source, a monitor, a remote control, and a central processing unit in a single mobile cart for surgical guidance in clinical settings [18]; the FLARE system (Frangioni Laboratory, Boston, MA) consists of two monitors, light sources, a color video camera, and two cameras for simultaneous image acquisition at 700 nm and 800 nm respectively and has been deployed for various image-guided surgery applications [19, 20]. It. Despite these clinical and technical advances in NIRF imaging, most of the existing imaging devices are stand-alone systems that display intraoperative images on a separate screen. Using such system to display intraoperative information in an OR may interfere with the surgical procedure because they force surgeons to switch the view fields instead of focusing on the surgical field. In addition, existing intraoperative fluorescence imaging systems are typically bulky and may not fit in the limited OR space in a clinical setting. Furthermore, some imaging systems can only display fluorescence emission and surgical background sequentially, making it difficult for surgeons to correlate the screen display of surgical margin information with that of the actual surgical field.

Recently, wearable navigation devices have been developed for intraoperative assessment of surgical margins [21–23].These navigation devices integrate excitation light source, fluorescence filter, camera, and display in a wearable headset, allowing surgeons to evaluate surgical margins intraoperatively in a natural mode of visual perception. Since these wearable devices are based on head-mounted display (HMD) or other wearable visual aids, they are sometimes cumbersome to surgeons and may easily block their visions. Furthermore, the surgical margin images are only visible for the individual who wears the visual aid but inaccessible for others in the OR without using an additional screen display. Finally, image fusion between background and fluorescence images requires a time-consuming computational process and takes the risk of losing critical disease information due to the limited resolution of the visual aid and the overlapping features between background and fluorescence images. To overcome these limitations, researchers introduce a new approach to project the imaging data directly on the patient within the designated regions of interest, such as skin, internal organs, and bone [24]. These projection systems are typically bulky, complicated, and are not optimized for intraoperative delineation of tumor margin.

**Fig 1:**
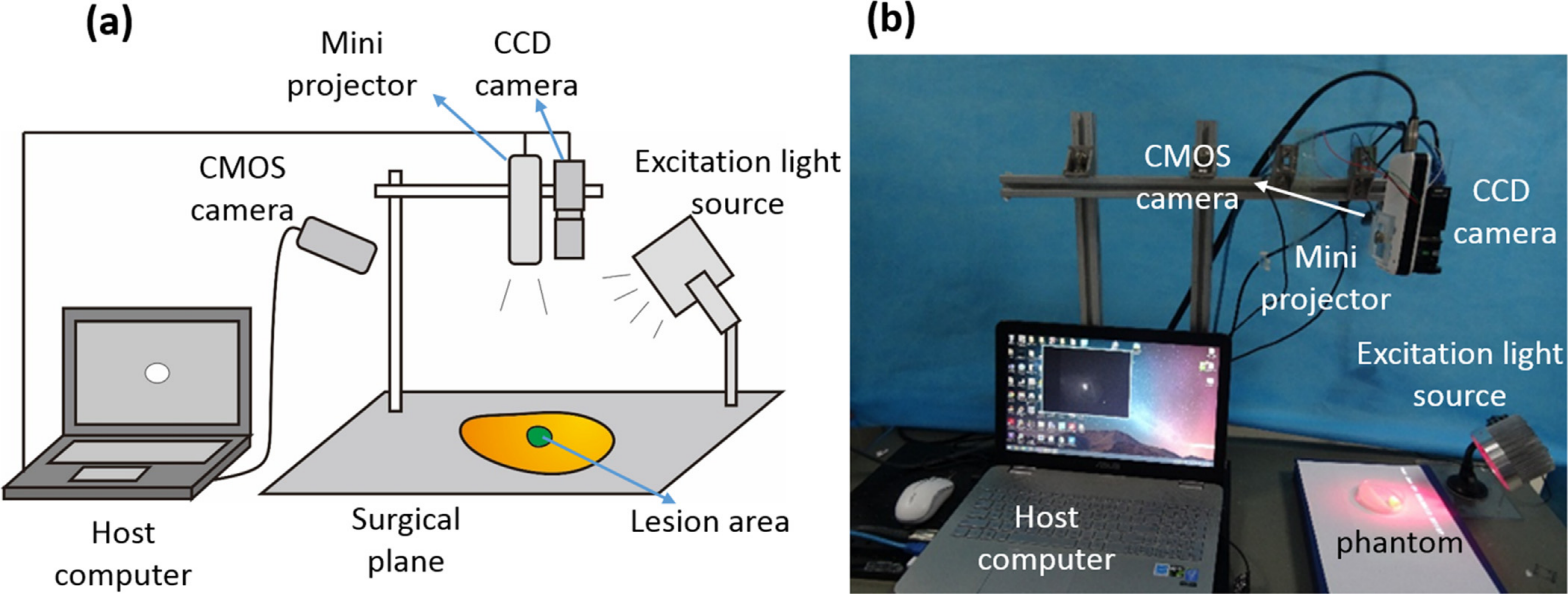
Fluorescence imaging projective surgical navigation system. (a) Schematic diagram of the projective surgical navigation system; (b) Projective surgical navigation system in working mode.

Here we describe a surgical navigation system that combines intraoperative fluorescence imaging and real-time surgical margin projection. The concept of this projective surgical navigation system is illustrated in Fig 1. Upon illumination of the surgical site by an LED array excitation light source, fluorescence emission is collected by a CCD camera and transmitted to a host computer. The acquired fluorescence images are processed and projected back to the surgical site in pseudo colors by a mini-projector so that fluorescence information originally invisible by naked eyes are overlaid on the surgical field and directly visible by surgeons. This surgical navigation system replaces the traditional screen display or HMD by a projector so that surgeons can navigate over the surgical field and identify lesion areas intuitively in a natural mode of visual perception with minimal distraction.

## Materials and Methods

### Hardware Design

As shown in Fig 1, the major hardware components of the proposed projective surgical navigation system include a MV-VEM033SM monochromatic CCD camera (Microvision, Xian, China) for fluorescence imaging, a mini projector (MEGO, Tianjin, China) for projecting the visible images, an LED array for excitation light illumination at 690 nm, an US-100 ultrasonic wave proximity sensor (Xinyuansheng, Shenzhen, China) for surgical field detection and a C310 CMOS camera (Logitech, Suzhou, China) for surgical field imaging. The excitation light source consists of 12 super bright 690nm LEDs (Xilan, Shenzhen, China) aligned in two concentric circles, with four LED beads in the inner circle and eight in the outer circle. The excitation light source is driven by a direct current (DC) power supply with voltage of 24V and current of 0.55A. The excitation light source is secured by a suction mount equipped with a universal joint so that the illumination distance and direction can be effectively adjusted. The excitation light source is placed 7 to 15 cm away from the surgical site and the illumination area is about 70 to 90 cm^2^. The CCD camera is installed 35 cm above the surgical field to acquire the fluorescence images at a frame rate of 120 frame per second (fps) and a resolution of 640×480. The CCD camera has a monochromic chip sensor of one-fourth inch with a dynamic range of 10 bits. A prime lens with a focal length of 5 mm is mounted on the camera in order to reach a field of view (FOV) of 224 mm ×168 mm for surgical observation. Between the CCD sensor and the lens, an FELH0800 800 nm long pass filter (Thorlabs Inc., Newton, NJ) is mounted so that fluorescence emission in the near infrared wavelength range is collected only. The DLP mini projector installed next to the CCD camera is used to project the processed images to the surgical field at a contrast ratio of 3500:1 and a resolution of 854 x 480. The projector consists of three-color RGB LEDs with a brightness of about 300 lumin, a projection area of 24.2cm × 13.7cm, and a projection distance of 35cm.The CMOS camera acquires the surgical field images for image registration tasks as described below in detail. A desktop computer is used to process the fluorescence images and transfer the processed images to the projector.

### Image Registration Algorithms

Considering that the projector and the CCD camera do not share the same optical path, calibration is necessary in order to ensure accurate mapping of the projected fluorescence images with the actual surgical scene. For this purpose, a set of four fiducial markers are placed at four corners of the surgical field to register the fluorescence images before projection. An automatic registration algorithm is implemented by calling Open Source Computer Vision Library in Microsoft Visual C++. A transformation matrix is derived based on perspective transformation to align the four projected fiducial markers with those in the surgical field. In a homogeneous coordinate system, a general form of transformation can be expressed as below:

$$\left(x^{\prime },{\text{y}}^{\prime },{\text{w}}^{\prime }\right)=\left(u,v,w\right)\;\left(\begin{array}{ccc} m_{11} & m_{12} & m_{13}\\ m_{21} & m_{22} & m_{23}\\ m_{31} & m_{32} & m_{33} \end{array}\right)$$

Where m11 to m33 are transformation parameters, (*u*, *v*, *w*) are original three dimensional coordinates, and (*x*′,y′,w′) are transformed coordinates. *w* and *w*′ are the additional scalar for homogeneous coordinate, which are usually generalized to one for points. For perspective transformation to an image, the following operation is carried out:

$$dst\left(x,y\right)=src\left(\frac{m_{11}x+m_{12}y+m_{13}}{m_{31}x+m_{32}y+m_{33}},\frac{m_{21}x+m_{22}y+m_{23}}{m_{31}x+m_{32}y+m_{33}}\right)$$

**Fig 2:**
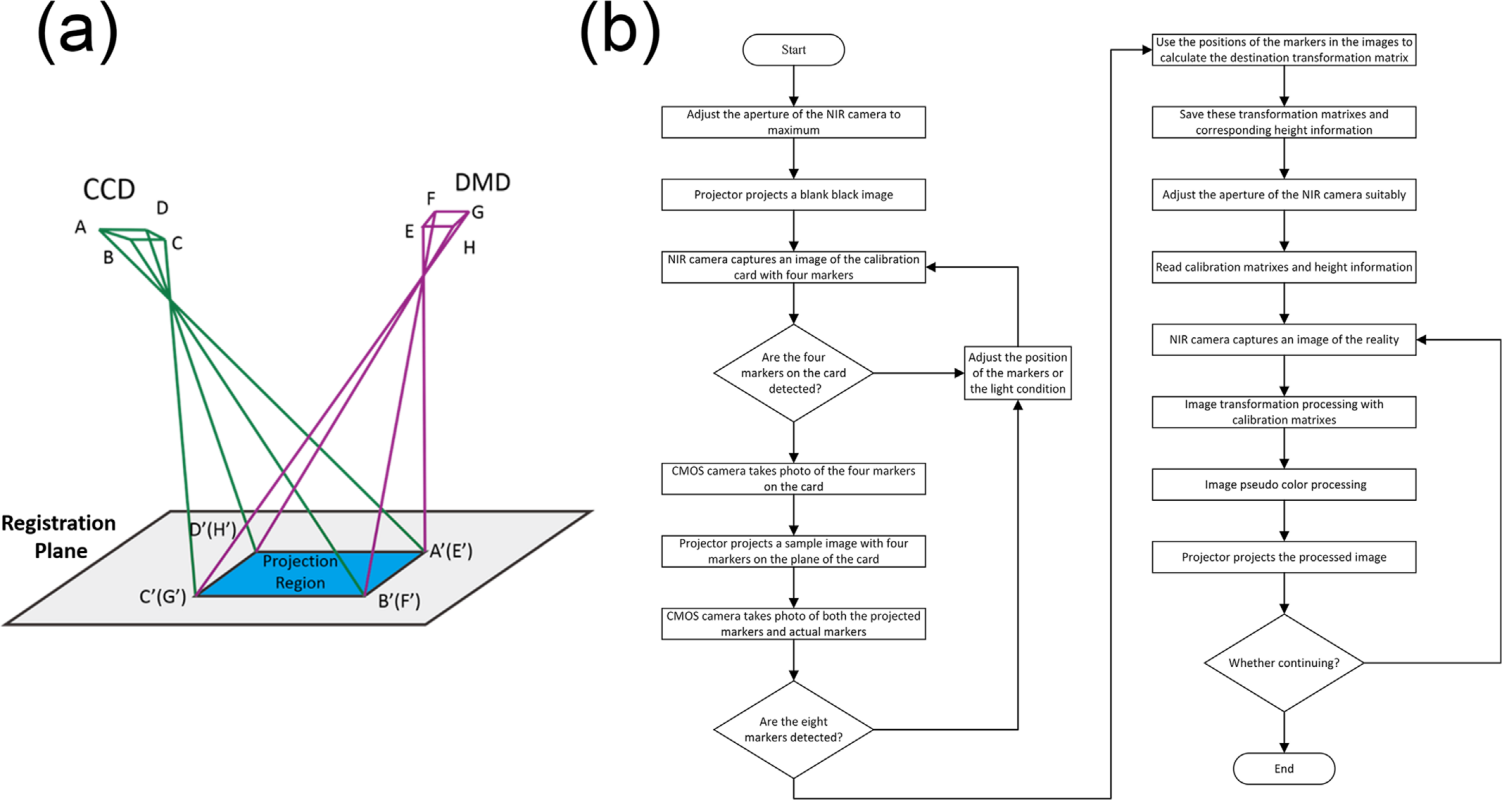
System registration method. (a) Schematic diagram of the system’s registration procedure; (b) Flow chart of the registration program.

Both the CCD camera’s optical path (between the CCD chip image ABCD and the surgical scene A’B’C’D’) and the DMD projector’s optical path (between the DMD chip image EFGH and the projected image E’F’G’H’ to the surgical scene) can be well described by perspective transformation, as shown in Fig 2A. When E’F’G’H’ is coincident with A'B'C'D', the projected image precisely overlays the actual surgical scene and the fluorescence image acquired by the CCD camera can be projected to the surgical scene to highlight the disease margin. Thus, to overcome projector distortion and ensure perfect match between the projected disease margin and the actual surgical field, a perspective transformation algorithm is used to convert image ABCD acquired by the CCD camera into image EFGH formed by the DMD so that the projected image to the surgical site coincides with the actual surgical scene. This process is therefore called “registration”. For effective registration of the projected images on the surgical plane, we identify the perspective transformation matrix by calibrating the system at four points mapping the source image (EFGH) with the target image (ABCD). To ensure one-to-one correlation at these four points, we place four fiducial markers at the corners of the surgical field, and project four corresponding fiducial markers on to the field for calibration. With the help of an extern CMOS camera, all perspective transformation between any two devices or images can be identified by these markers and their projections. The transformation, between the markers on DMD being projected, and the points on the DMD corresponding to the physical fiducial markers, is the perspective transformation that we are looking for. The transformation has a representative matrix denoted by tm.

Fig 2B shows the flow chart of our automatic registration program for system calibration. Before the calibration process, a piece of white paper with four fiducial markers is placed at the center of the surgical plane. The apertures of the NIR (CCD) camera and the CMOS camera are adjusted so that all the fiducial markers can be effectively detected by both cameras. The image of four fiducial markers acquired by the NIR camera is projected by the mini projector to the white paper. The CMOS camera then acquires an image of the surgical scene that consists of both the physical fiducial markers and the projected ones. Once all the eight markers are imaged and recognized, the automatic registration program will calculate the corresponding transformation matrix. Finally, image with fiducial markers captured by NIR camera is transformed by the matrix and projected to the white paper to evaluate the calibration accuracy. Such a process may iterate until perfect registration between the original and the projected images is achieved.

**Fig 3:**
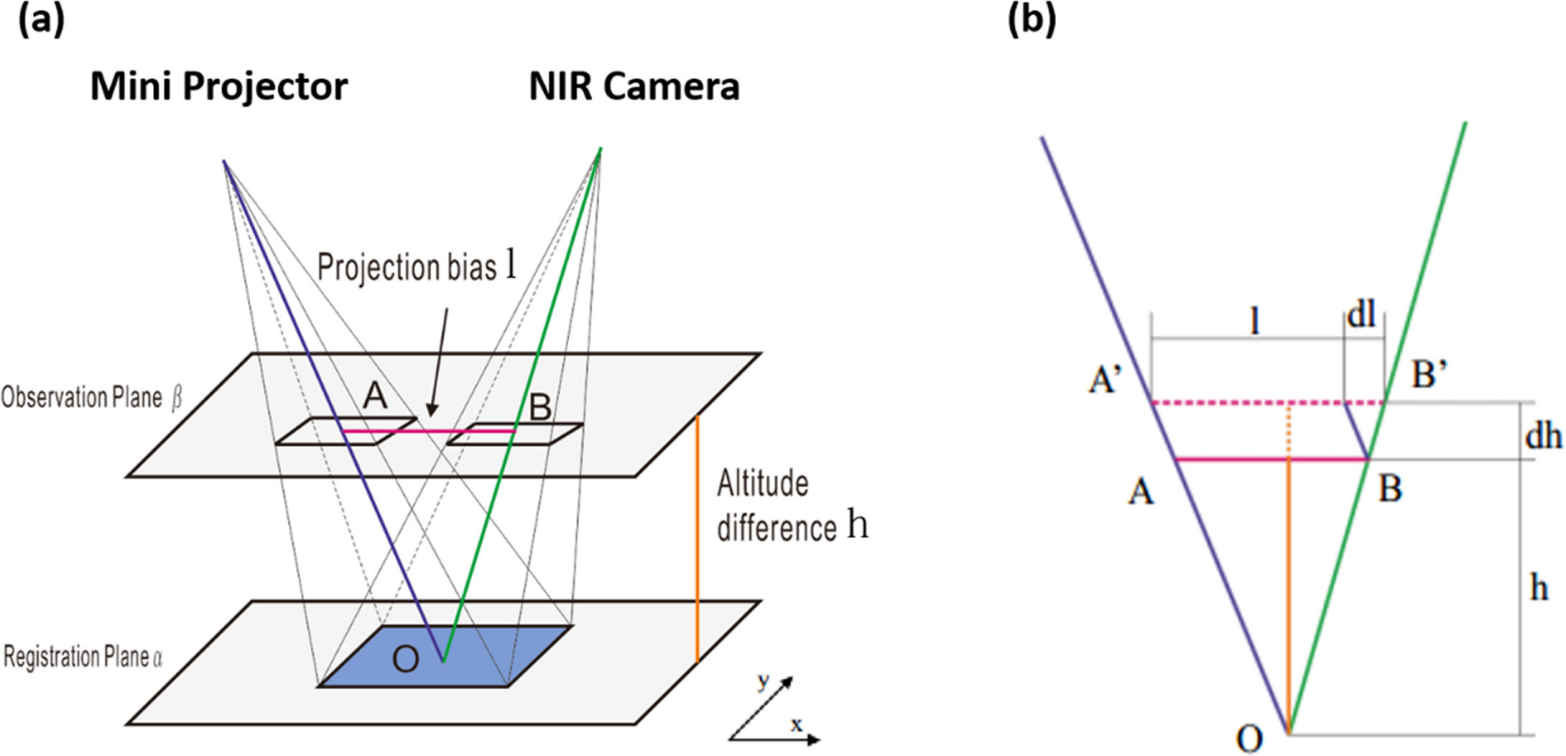
The relationship between projection bias and height difference. (a) Schematic diagram of the projection bias induced by height variation after registration, plane α is the registration plane and there is a bias l on the observation plane β; (b) The triangle caused deviation in (a).

After the above automatic registration process, the projected images and the actual objects are coincident with each other on the registration plane (i.e., the surgical plane). However, according to Fig 3A, as height h of the surgical plane changes, the projected image may shift by a distance l since the optical axis of the projector is not aligned with that of the NIR camera.

Fig 3B show the geometric illustration of the height-induced projection bias. The elevation of the surgical plane by dh increases the projection bias by dl. In plane AOB, triangle AOB and A’OB’ are similar triangles. Therefore, the relative change of projection bias l is linearly proportional to the relative change of the imaging height h.

$$\frac{h}{l}=\frac{h+dh}{l+dl}$$ (1)

$$K=\frac{dl}{dh}=\frac{l}{h}$$ (2)

K in Eq (2) is defined as a slope factor for height-induced projection bias. As bias l can be decomposed into lx and ly in x and y directions, K can also be decomposed into Kx and Ky, respectively:

$$K_{x}=\frac{dl_{x}}{dh}=\frac{l_{x}}{h}$$ (3)

$$K_{y}=\frac{dl_{y}}{dh}=\frac{l_{y}}{h}$$ (4)

Through the above analysis, it is clear that the height-induced projection bias can be effectively corrected as long as the height difference h and the slope factors Kx and Ky are known in advance. For this purpose, we propose a method to compensate for the projection bias induced by the height difference of the surgical field and the optical path difference between the projector and the camera.

This method is best illustrated by Fig 3A where two projection planes with height difference h are presented. The compensation is done in two steps. First, the slope factors are calculated based on the transformation matrices of these two projection planes tm1 and tm2 respectively. For any point (*x*, *y*, 1) on the fluorescence image, the transformed points are given by Eqs (5) and (6), and the projection bias AB can be obtained by Eq (7), where the transformed points are generalized. The additional scalar for the coordinate for AB is zero, indicating it is a line segment:

$$(x_{1},y_{1},w_{1})=(x,y,1)\cdot tm_{1}$$ (5)

$$(x_{2},y_{2},w_{2})=(x,y,1)\cdot tm_{2}$$ (6)

$$AB=\left(l_{x},l_{y},0\right)=\frac{1}{w_{1}}(x,y,1)\cdot tm_{1}-\frac{1}{w_{2}}(x,y,1)\cdot tm_{2}$$ (7)

Second, the slope factors *Kx* and *Ky* are calculated from Eqs (3) and (4) separately with *AB* and *h*. As the height of the observation plane (i.e., *h*) changes, the projection bias in *x* and *y* directions (i.e., *l*_*x*_ and *l*_*y*_) are calculated and the source images are translated accordingly so that the destination images coincide with the actual scene without the need for additional calibration.

$$dst\left(x,y\right)=src\left(x-k_{x}h,y-k_{y}h\right)$$ (8)

In the case of non-planar working surface, the image is divided into pixels and regarded as individual small planes for pixel by pixel correction of height. After the whole image is transformed with the perspective transformation tm1, the position of each pixel point (*x*, *y*) with individual relative height *h* to the registration plane, is modified using Eq (8) separately. After all the pixels are processed, the destination image would coincide with the actual scene as it is projected.

### Performance Validation and Optimization

The imaging performance of the proposed projective surgical navigation system is characterized and optimized by benchtop experiments using Indocyanine green (ICG) as a fluorescence contrast agent [23]. ICG is a cyanine dye approved by US Food and Drug Administration (FDA) for fluorescence imaging in many medical diagnostic applications. In the arena of cancer management, ICG has been used to localize the sentinel lymph nodes (SLNs) in many cancers, such as breast cancer[9], skin cancer[25], superficial esophageal[26] and gastric cancer[27]. In aqueous solution, ICG tends to aggregate and shifts its peak absorption wavelength from about 780 nm to about 700 nm, depending on its concentration, while its fluorescence emission spectrum peaks at around 820 nm [28]. After intravenous injection, the main peak absorption wavelength of ICG shifts to 805 nm owing to polymerization and aggregation to plasmatic proteins, while its fluorescence emission peak experiences multiple variations, depending on different phase after injection [28].

The intensity of the fluorescence emission is directly related to the excitation light. High fluence rates run the risk of heating tissue and causing thermal damage. When the excitation fluence rate is >50 mW/cm², small-molecule organic fluorophores typically undergo irreversible photobleaching [17]. A range of 0–20 mW/cm² is applicable for excitation light intensity [21]. We measure our system’s excitation light source using a VEGA optical power/energy meter (Ophir, Jerusalem, Israel). The distances between the power sensor and the light source are 7–17 cm. The mean intensities are 4.37–6.57 mW/cm². The maximum value is about 9 mW/cm², which is less than 50 mW/cm².

**Fig 4:**
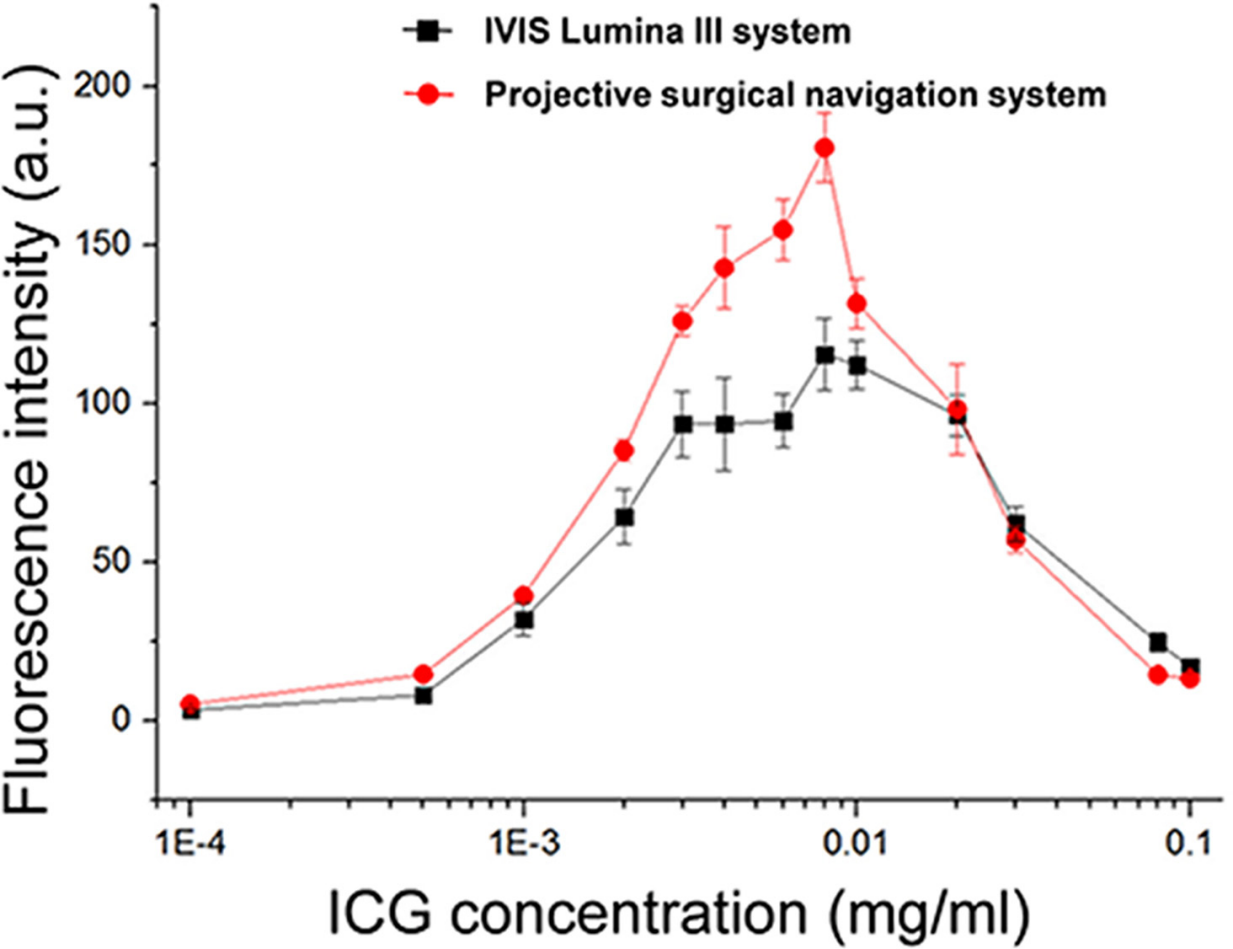
Comparison of fluorescence detection capability. The concentration of ICG solution are 0.0001, 0.0005, 0.001, 0.002, 0.003, 0.004, 0.006, 0.008, 0.01, 0.02, 0.03, 0.08, 0.1 mg/ml respectively. The black line is the detection results by IVIS Lumina III and the red line is by the NIR Camera in our projective surgical navigation system.

The performance characteristics of the proposed projective surgical navigation system are tested using aqueous solution of ICG (BOMEI pharmaceutical, Shenyang, China) at different concentrations and compared with those of a commercial IVIS Lumina III small animal imaging system (Perkin Elmer, Waltham, MA).Thirteen ICG samples with concentration levels ranging from 0.0001 mg/ml to 0.1 mg/ml are prepared in advance. Each sample is divided into two parts for fluorescence imaging using our system and the small animal imaging system respectively. At each concentration level, the imaging experiments are repeated four times and the fluorescence intensity levels are averaged and plotted, as shown in Fig 4. According to the figure, the proposed projective surgical navigation system has a comparable if not better sensitivity and dynamic range for detecting fluorescence emission in aqueous solution of ICG.

**Fig 5:**
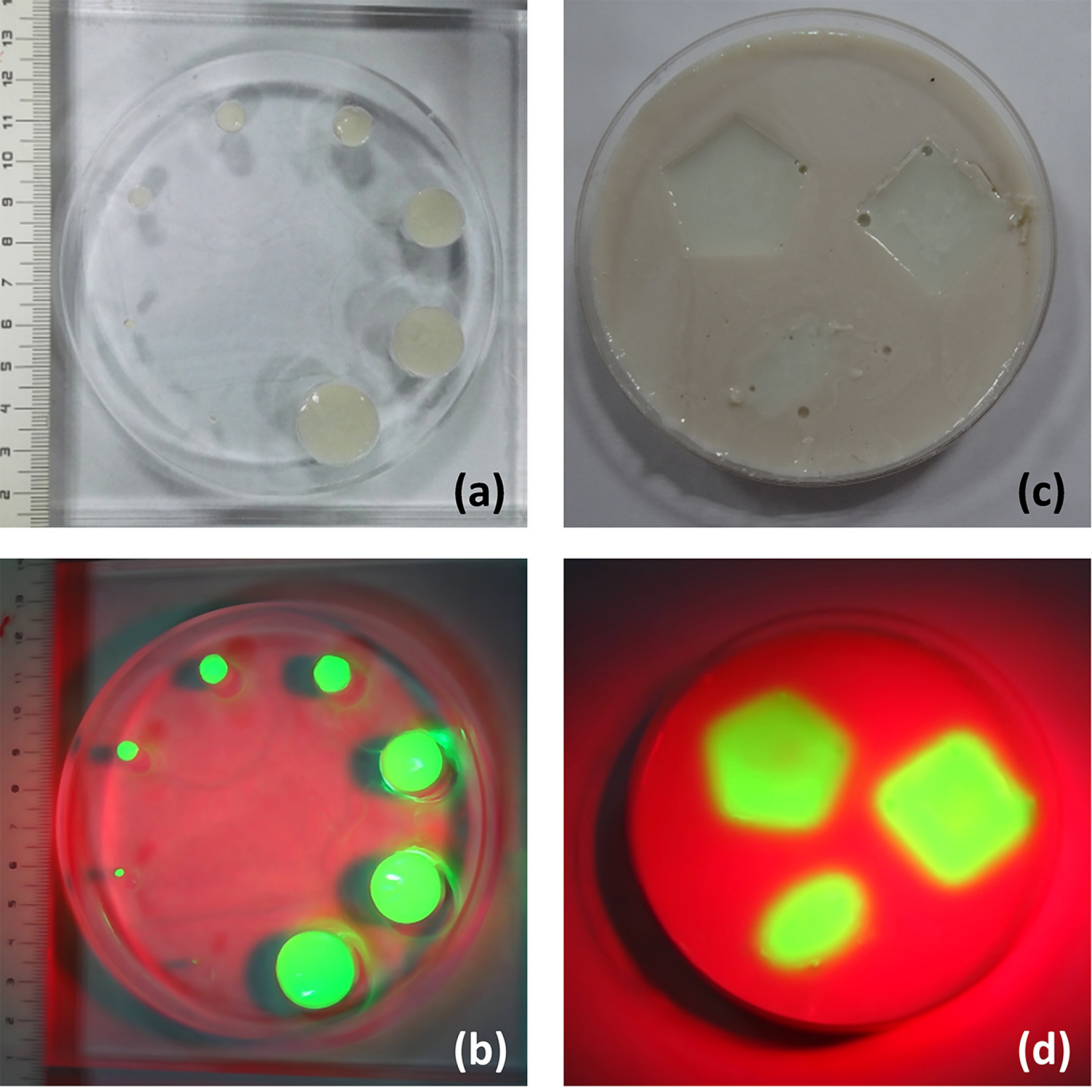
Phantoms that embed agar-agar gel tumor simulators for validation of projective surgical navigation system. (a) A PMMA phantom embedding agar–agar gel cylinders of different diameters from 1 mm to 20 mm; (b) Fluorescence emission of the agar-agar gel cylinders is effectively acquired and projected to the phantom by our projective navigation system; (c) An agar–agar gel phantom embedding agar-agar gel nuggets of different shapes; (d) Fluorescence emission of the agar-agar gel nuggets is effectively acquired and projected to the phantom by our projective navigation system.

The capability of our projective surgical navigation system for acquiring and projecting fluorescence images is demonstrated in a series of agar-agar gel and PMMA phantoms. Nuggets of different shapes and sizes are made by ICG-loaded agar-agar gel and embedded in these phantoms to simulate different tissue anomalies. Fig 5A shows a PMMA phantom that embeds cylindrical tumor simulators from 1 mm to 20 mm. To prepare this phantom, 0.56mg ICG, 2.1g Agar, 0.28g Titanium dioxide, 4.9ml Glycerol and 70ml distilled water are mixed, heated to 90°C, stirred by magnetic stirrer for 30–40 minutes in a beaker, poured into the holes drilled in a PMMA disk, and cool down for 30–40 minutes. The diameters of the holes are 1mm, 2.5mm, 5mm, 7.5mm, 10mm, 15mm, 17.5mm and 20mm, respectively. Fig 5C shows an agar-agar gel phantom that embeds the nuggets of ICG-loaded agar-agar gel with different shapes. The nuggets are prepared in advance following the same recipe as that of the PMMA phantom. As shown in Fig 5B and 5D, our projective navigation system effectively identifies the positions and the shapes of the tumor simulators, while the surrounding phantom materials do not show fluorescence emission.

As we have discussed before, the projected image by our projective surgical navigation system may deviate from its physical position if the height of the projection plane changes. This deviation is partially caused by inconsistent optic paths between the projector and the CCD camera, and can be calibrated in advance at different heights. For this purpose, a series of experiments are designed to measure the height-induced projective bias and to demonstrate the effectiveness of our bias compensation algorithm as described before. Before the experiment, a tumor-simulating phantom is prepared by inserting two ICG-loaded agar-agar gel cylindrical chips in a large base of agar-agar gel, with the boundary of the cylindrical chips marked for visibility. The phantom is placed on a projection plane driven by a motorized linear stage (Zolix, Beijing, China). The excitation light source and the CMOS camera are also installed on the linear stage with fixed positions relative to the projection plane. At the beginning of the experiment, the CCD camera is placed 20 cm above the projection plane and this plane is marked as the reference plane with height h = 0. As the projection plane is driven by the linear stage to move from h = -50 mm to 50 mm at a step size of 5 mm, the background and the fluorescence images are acquired at each step and projected back to the projection plane. For automatic compensation of height-induced projection bias, an US-100 ultrasonic sensor is installed beside the CCD camera to detect the height offset of the projection plane from the reference plane. At each position of the projection plane from -45 mm to 45 mm (5 mm per step, totally 19 positons), the actual height of the projection plane is detected by the ultrasonic sensor and sent to the host computer for automatic correction of the height-induced projection bias.

We also demonstrate the technical feasibility of automatic compensation of height-induced projection bias simultaneously in different projection planes. Four planes of different heights (i.e., A: 0 mm, B: 20 mm, C: 65 mm and D: 115 mm) are used in the experiment. Plane A is chosen as the reference plane and the projective navigation system is calibrated on this plane in advance. Heights of the rest three projection planes are sent to the computer for automatic compensation of the projection bias.

We further demonstrate the surgical margin projection technique in an ex vivo chicken tissue model. To prepare this ex vivo model, we prepare the tumor simulator by mixing 0. 56 mg of ICG, 2.1 g of agar, 0.28 g of titanium dioxide, 4.9 ml of glycerol and 70 ml of distilled water at 90°C, inject the mixture into a piece of fresh chicken breast tissue at an estimated depth of 1 mm, and let it cool down and solidify for about 30 minutes. As this ex vivo phantom is exposed to the excitation light, fluorescence emission from the tumor simulator is acquired by our projective surgical navigation system and compared with that acquired by the IVIS small animal imaging system.

### Animal Experiments

A BALB/c-nu nude mouse (NU/NU, Vital River Laboratory Animal Technology Co. Ltd, Beijing, China) at 7-week-old and with the weight of about 20 g is used for the animal validation test. The animal protocol is approved by the Institutional Animal Care and Use Committee of University of Science and Technology of China (protocol No: USTCACUC1501014). Before the experiment, animals are housed at the Specific Pathogen Free (SPF) feeding room in the Core Facility Center for Life Sciences, USTC. As mentioned in the animal protocol, the animal is anesthetized by intraperitoneal injection of chloral hydrate (350mg/kg). The total ICG dose injected should be kept below 2 mg/kg. The injected dosage is calculated on the basis of 0.5 mg/kg (ICG drug instructions from the pharmaceutical company (Akorn, Lake Forest, IL, USA), which has FDA certification).ICG is dissolved in sterile water in advance to make a total volume of 0.5–1 ml and concentration of 0.01 mg/ml. After anesthesia, the animal is fixed on a surgical panel in a supine position, with four limbs stretched out and secured by medical tapes. During the experiment, the animal condition is closely monitored every 5 minutes to ensure that the test procedure introduces no pain or stress. The excitation light source, the CMOS camera, and the CCD camera are placed above the surgical plane with appropriate adjustment for effective illumination and imaging acquisition from the whole animal. After a tail vein bolus injection of ICG solution at a dose of 0.4 mg/kg body weight[29–31], the fluorescence images are acquired, processed by the host computer, and projected back to the animal continuously until the end of the experiment.

## Data Analysis and Results

### Spatial Resolutions

**Fig 6:**
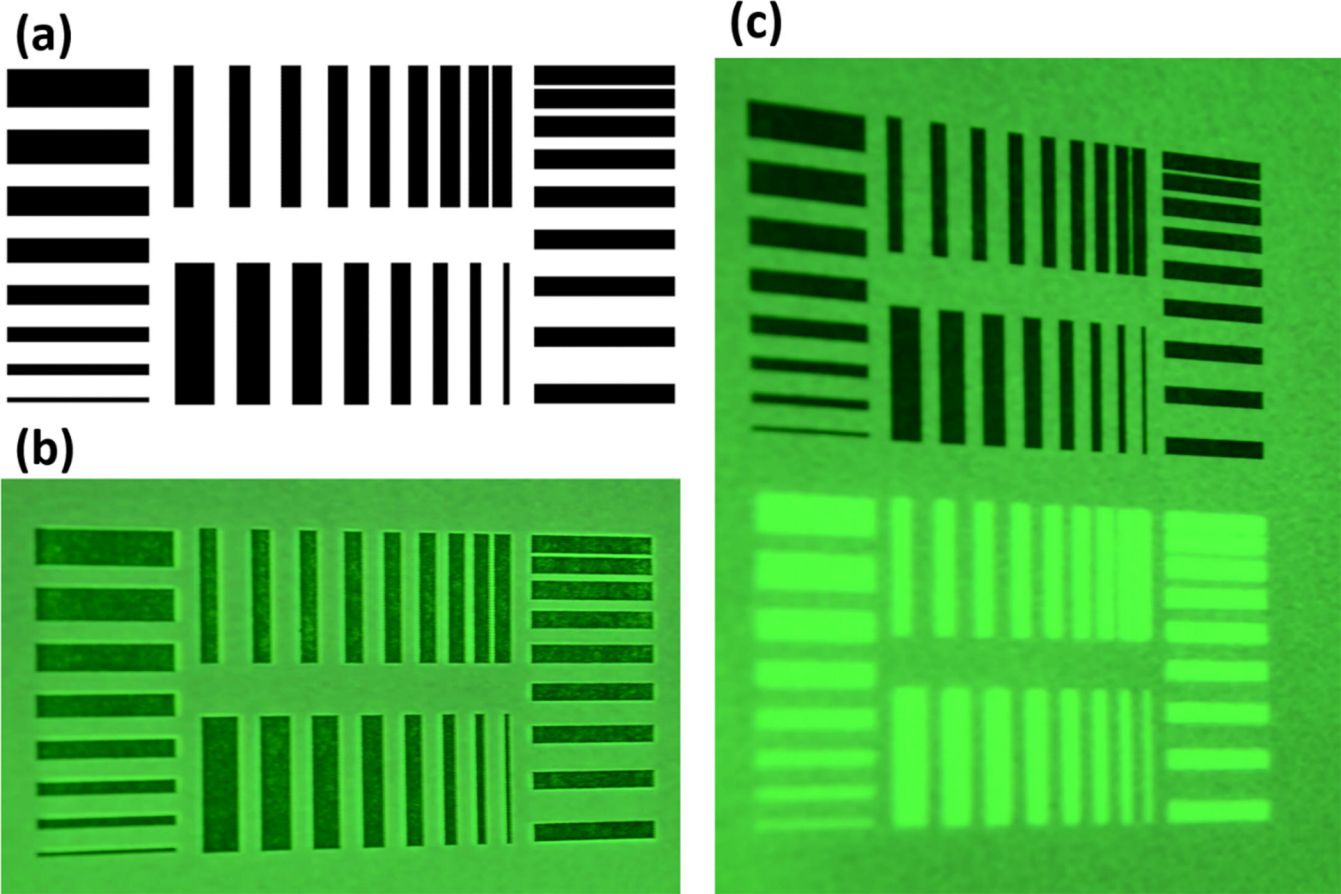
The achievable spatial resolutions of the projective navigation system. (a) The test pattern is 71 mm×36 mm, consisting of horizontal and vertical stripes with thicknesses from 0.5 to 4 mm and the separation distances from 0.5 to 4 mm. (b) The projected image overlaid with the printed test pattern. (c) Side by side comparison between the test pattern and the projected image.

The achievable spatial resolutions of the projective surgical navigation system are tested using a test pattern as shown in Fig 6A. The size of the test pattern is 71 × 36 mm and consisted of horizontally and vertically printed stripes. The stripes are divided into two groups. In one group, the stripes’ thicknesses are 0.5, 1.0, 1.5, 2.0, 2.5, 3.0, 3.5, and 4.0 mm with the same separation (2 mm). In another group, the stripes are separated by 0.5–4.0 mm, with each 0.5 mm increase per stripe. According to Fig 6B, the projected stripes overlay the printed stripes on the paper, making it difficult to distinguish and quantitatively analyze the resolution. Therefore, we program the system to project the processed image to the side of the printed stripes with clear separation. As shown in Fig 6C, features smaller than 0.5 mm could be captured by the naked eye in both vertical and horizontal directions.

**Fig 7:**
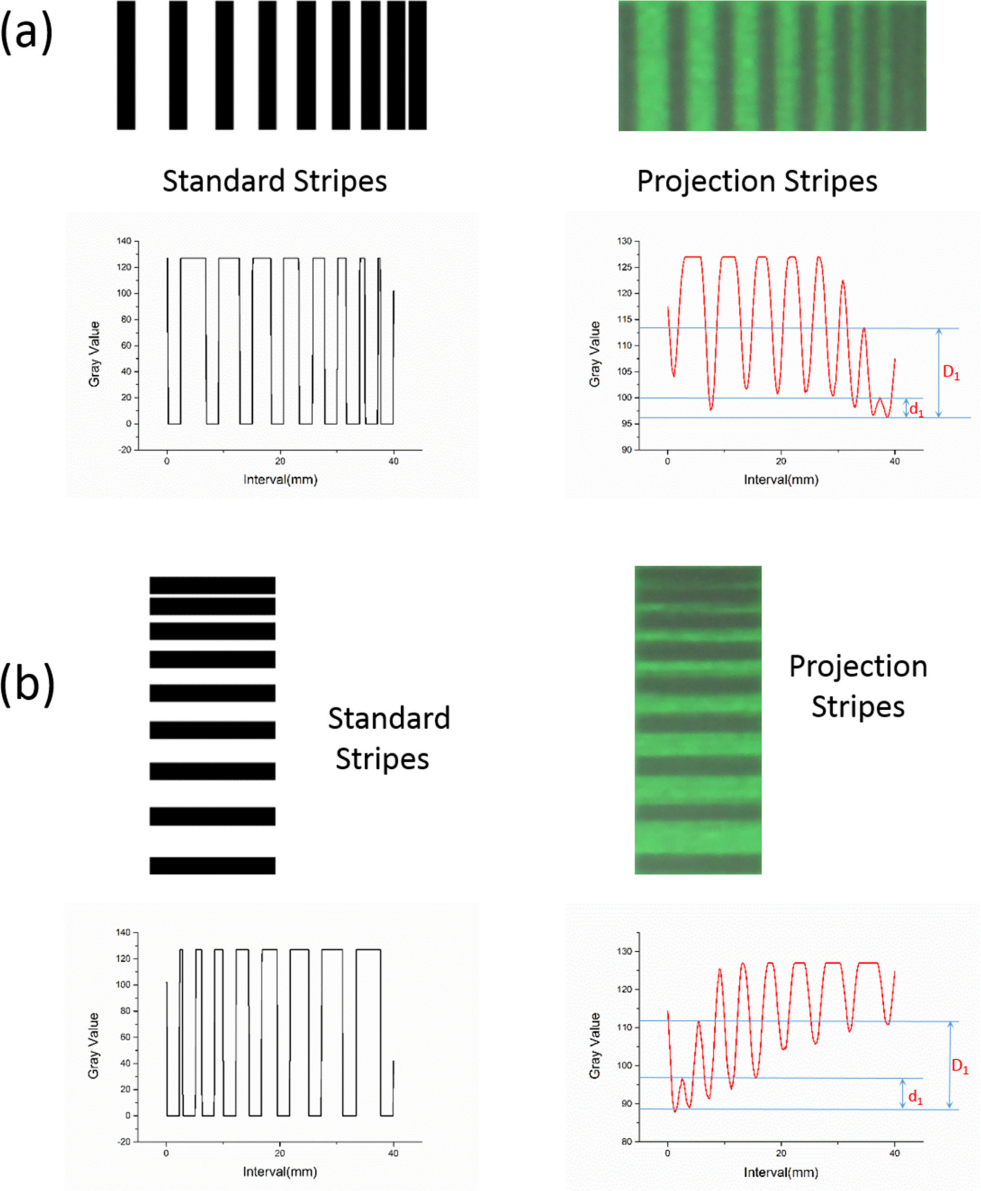
Individual analysis of the spatial resolutions’ test pattern. (a) Variable separation of stripes in the horizontal direction. (b) Variable separation of stripes in the vertical direction.

The stripes, separated with different intervals in the test pattern, are extracted for individual tests. The projection stripes are compared with standard stripes. All images are converted to grayscale. The gray values of the stripes’ middle points are then calculated and plotted as a line graph. As shown in Fig 7A and 7B, Δ1 = d1/D1 = 0.212 < 0.263 and Δ2 = d2/D2 = 0.326 > 0.263. According to the Rayleigh criterion[32], the differentiable separation is less than 0.5 mm in vertical direction and is within the range of 0.5–1.0 mm in the horizontal direction.

### System Response

**Fig 8:**
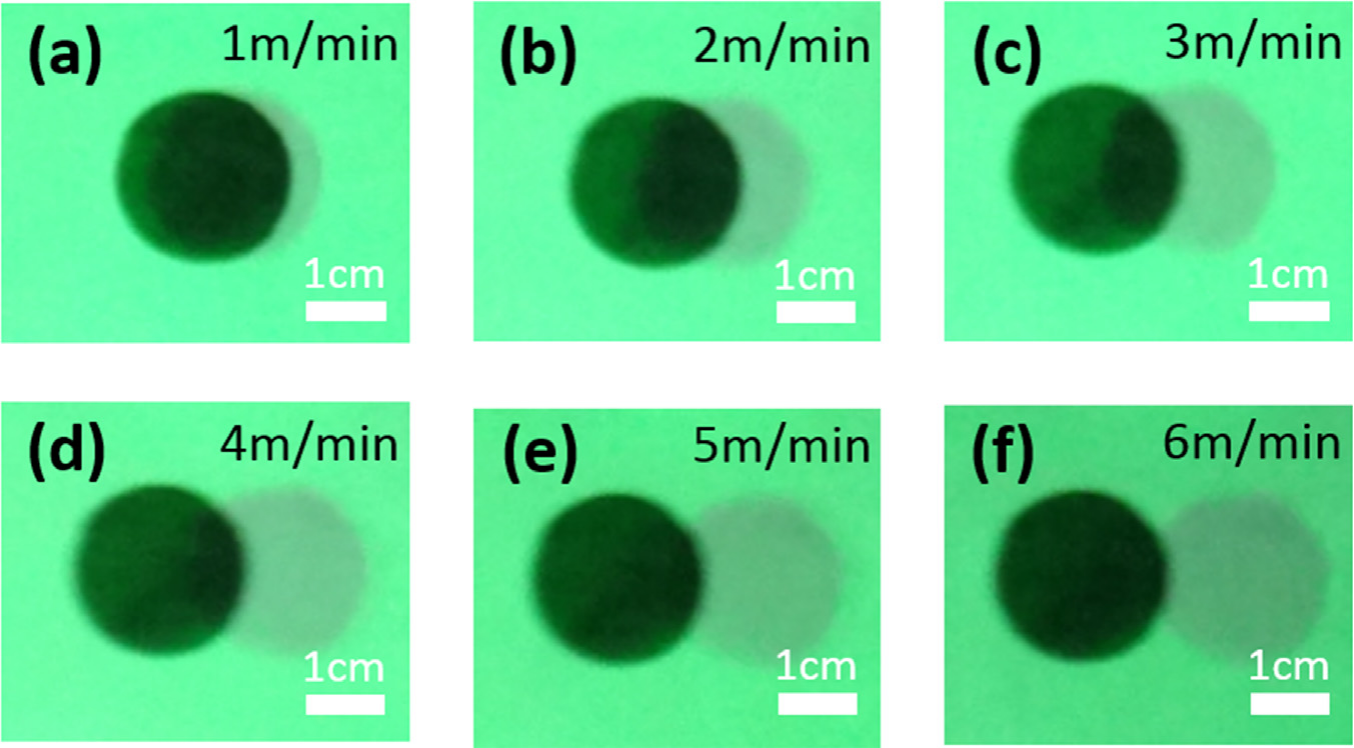
Experimental results that simulate the effect of the surgical scene motion on the imaging lag of the projective navigation system. (a-f) show the projected pattern (gray dot) and the actual object (black dot) at the following moving speeds: 1, 2, 3, 4, 5 and 6 m/min, respectively.

Considering the time required to acquire, process and project the surgical field images, it is expected that the projective navigation system may experience a certain level of delay, especially when it is used for surgical navigation at a high speed. In order to estimate the system delay quantitatively, we print a black dot of 2 cm in diameter on a piece of paper and place the paper on a linear translational stage. After the system is calibrated, the linear stage is programmed to move horizontally at stepped speed levels from 1 m/min to 6 m/min, with 1m/min increase per step. At each speed level, the black dot is imaged by both the CMOS camera and the CCD camera and projected back to the paper after perspective transformation and bias compensation. Fig 8 shows the printed and the projected black dots at different speed levels. Based on these images, it is estimated that the overall delay time of the system is about 330 ms. Obviously, the projected image deviates more from its actual location as the moving speed of the surgical field increases. In order to reach a navigation accuracy better than 2 mm, the relative motion between the surgical field and the projective navigation system should not exceed the speed of 1 m/min. Considering that the adult respiration rate is 12–18 breaths per minute, corresponding to 3-5s per breath[33], the system latency of 330ms will not induce significant localization artifact owing to breathing movements. Considering that the projective surgical navigation system is installed above the surgical field instead of being worn by a surgeon, it is relatively easy to control the motion of the system within this speed level. Further software design and engineering optimization is necessary in order to shorten the system delay time for real-time projection without lag.

### Phantom Study

**Fig 9:**
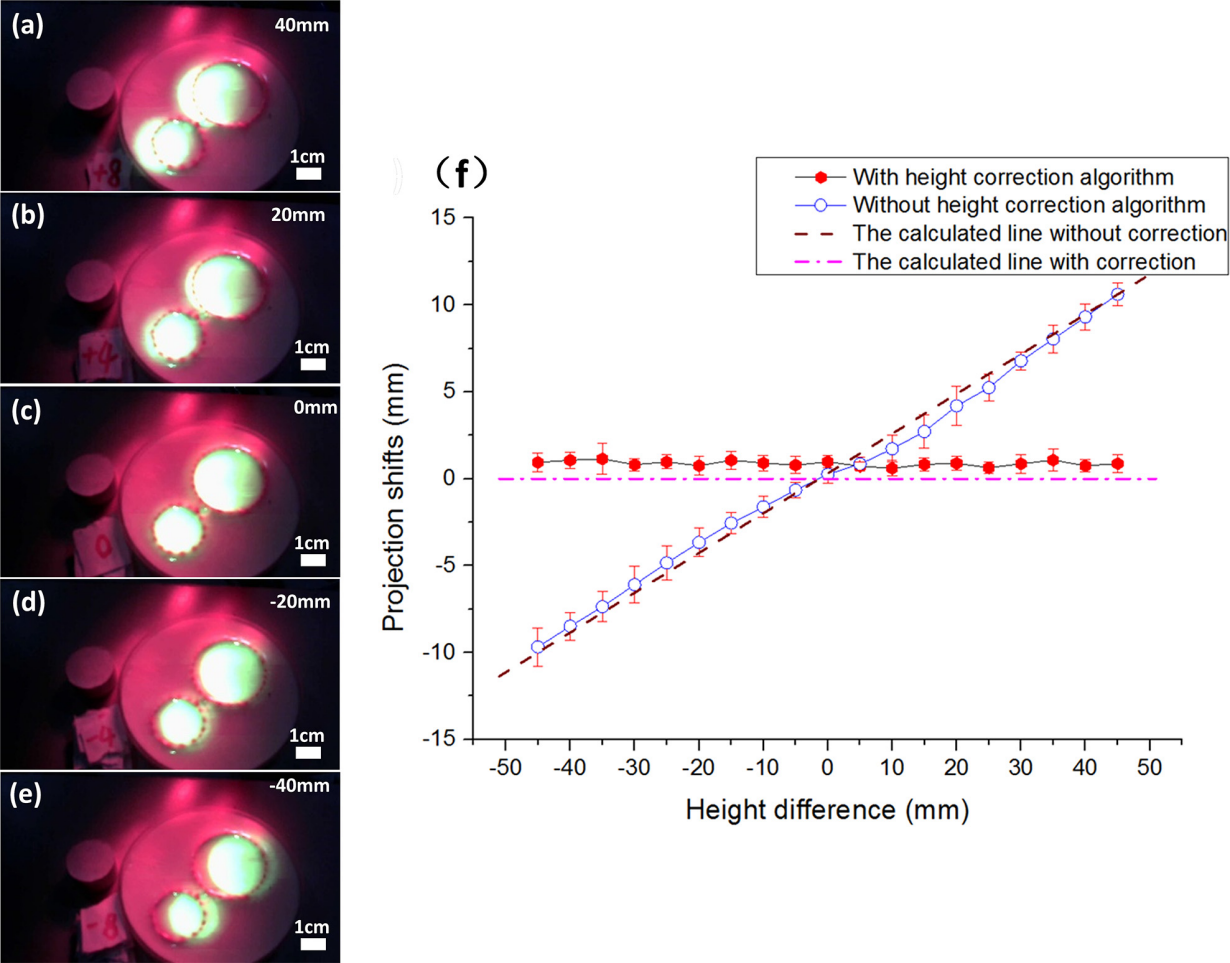
Phantom study to show the projective accuracy as the function of the height difference between the projection plane and the reference plane. (a)—(e): As the projection plane moves from 40 mm above to 40 mm below the registered reference plane, the projected image is biased from left to the right of the actual phantom. (c): If the projection plane is identical to the reference plane, the projected image and the actual phantom are co-registered without bias. (f) Linear relationship is observed between the projection bias and the height difference without height correction, while the projection bias is controlled within 1 mm for all the height differences after the height correction algorithm is applied.

As described before, the projected image may deviate from the actual position of the surgical scene when the height of projection plane is changed. Through geometric analysis as shown in Fig 3, we also hypothesize that the projection bias is linearly correlated with the height of the projection plane. This hypothesis is further validated through a series of experiments as shown in Fig 9. As the projection plane moves to different heights from -45 mm to +45mm, the CCD camera acquire the fluorescence image of two ICG-loaded cylindrical samples and projected it back to the projection plane, as shown in Fig 9A–9E. According to the figure, the projected image matches perfectly with the sample geometry at h = 0 mm, which is the calibrated reference plane. As the height of the projection plane deviates from the reference plane, the projection bias increases. In this study, the height of the projection plane is detected by a proximity sensor and calculate the transformation matrix for automatic compensation of height-induced projection bias. Fig 9F shows the averaged projection biases at different height levels before and after compensation. According to the figure, the projection bias is linearly correlated with the height of the projection plane before compensation algorithm is applied. After compensation, the projection bias is controlled less than 1 mm at any height and no longer had a liner relationship with the height differences, which prove that the relationship between the height differences and the offsets is liner and the height correcting algorithm is effective.

According to Fig 9, the projected image register well with the actual phantom objects only when the registration plane is on the reference plane where the system is calibrated in advance. As the registration plane deviates from the reference plane, a projection bias is induced and the bias level is proportional to the height difference between these two planes, as shown in Fig 9A–9E. Fig 9F shows that the projection bias is linearly correlated with the height difference. The figure further shows that the bias compensation algorithm can effectively eliminate the height-induced projection bias.

**Fig 10:**
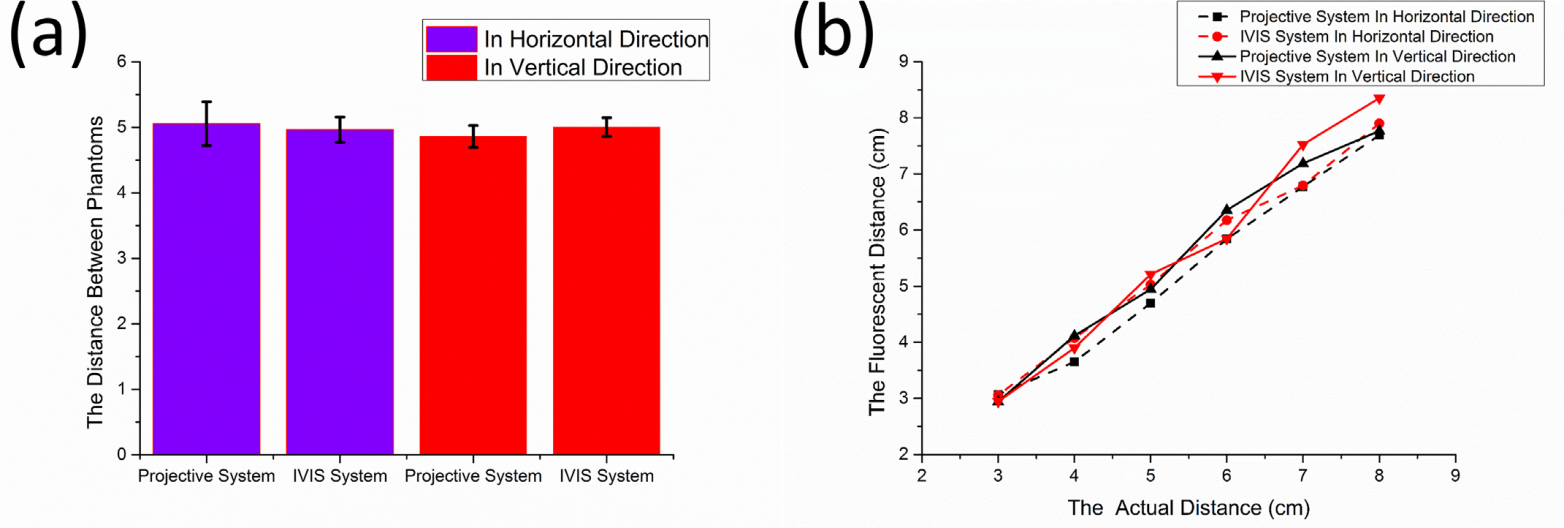
Comparison of the projective and IVIS systems in a phantom study. (a) Comparison of phantoms with different sizes for the same distance. (b) Comparison of phantoms with the same sizes at different intervals.

Our projective navigation system is compared with the IVIS small-animal imaging system using five sets of circular phantoms with different diameters (2.4, 1.9, 1.6, 1.3, and 0.9 cm). Each set includes three phantoms of the same size placed as a right-angled triangle with side lengths of 5 cm. For each set of phantoms, fluorescence images are acquired by both the projective system and the IVIS system in order to calculate the distances between the neighboring circular phantoms. Fig 10A compares the averaged phantom distances and the standard deviations measured by the projective system and the IVIS system in both the horizontal and the vertical directions. According to the figure, the p values of these measurements are greater than 0.05, indicating that the distances measured by our projective system have no significant difference from those by the IVIS system. In another experiment, fluorescence images of a set of phantoms with the same sizes but separated by different intervals (3, 4, 5, 6, and 7 cm) are captured by the two systems. According to Fig 10B, the intervals detected by the two systems were closely approximated, and the results had the same tendency in both directions.

### Ex-vivo Study

**Fig 11:**
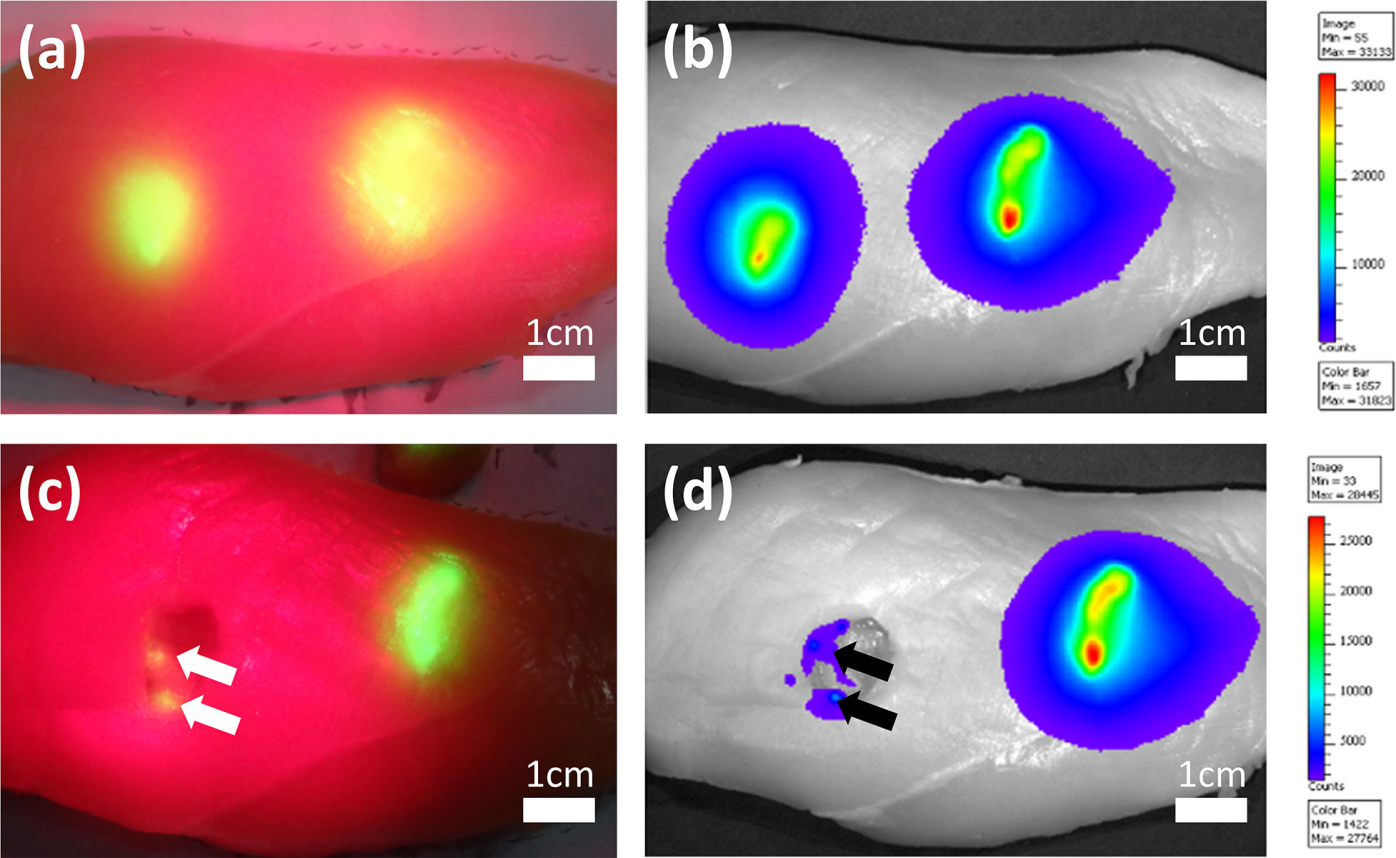
Demonstration of the image-guided tumor resection procedure using an ex-vivo tumor simulating tissue model. (a) Tissue model observed by the projective navigation system before resection. (b) Model observed by the IVIS small animal imaging system before resection. (c) Model observed by the projective navigation system after resection. Arrows correspond to the identified residual lesions. (d) The IVIS system also captures two residual lesions (black arrows).

An ex-vivo tumor simulating tissue model as shown in Fig 11A is used to evaluate the performance of the projective navigation system and demonstrate the image-guided tumor resection procedure. Fresh chicken breast tissue is injected with ICG-loaded agar-agar gel solution and placed in the surgical field. As the excitation light at 690 nm illuminates the chicken tissue, fluorescence emission from the injected ICG-loaded agar-agar gel solution is acquired by our navigation system and projected back to the surgical scene in a pseudo color, as shown in Fig 11A. Under the guidance of the projected pseudo color image at the surgical site, we are able to complete the surgical resection procedure without the need for looking at the screen display. Further, the projection navigation system enables us to localize the residual lesions in the surgical cavity for re-resection, as shown by the arrows in Fig 11C. To evaluate the imaging performance of our projective navigation system, the same chicken tissue is also imaged by an IVIS Lumina III small animal imaging system (Perkin Elmer, Waltham, MA) before and after resection. According to Fig 11B and 11D, the IVIS system shows the surgical margins and the residual diseases coincident with those by the projective navigation system, indicating the technical feasibility for intraoperative navigation and residual lesion detection in cancer resection surgeries.

### In-vivo Study

**Fig 12:**
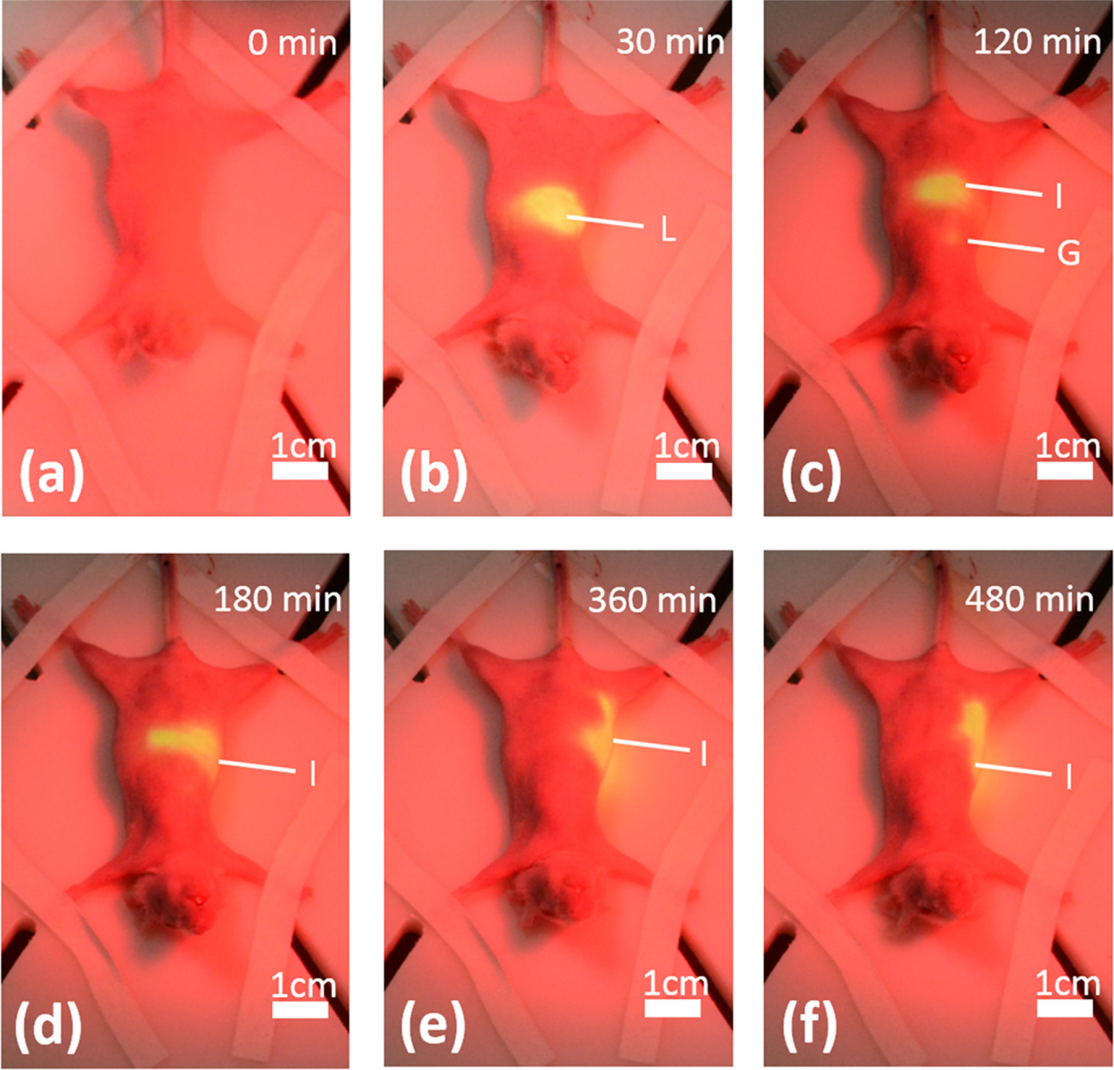
In vivo distribution of ICG in nude mouse after tail vein injection. (a-f) The images using projective navigation system at various times pose intravenous injection of ICG. Image acquisition times are 0, 30 minutes, 2 hour, 3 hours, 6 hours, 8 hours after injection separately. Images show dye accumulation in the liver(L), gall bladder(G) and intestines(I).

The projective surgical navigation technique is also demonstrated in vivo in a nude mouse model. Fig 12 shows a series of fluorescence images projected in pseudo color at 30, 120, 180,360, and 480 mins after tail vein bolus injection of ICG solution at a dose of 0.4 mg/kg body weight. Using the projective navigation system, the pharmacokinetic characteristics of ICG can be directly visualized by naked eyes. Within 30 minutes after tail vein injection of ICG, the fluorescence dye is quickly accumulated in the liver, leading to the increased brightness in the projected images, as shown in Fig 12B. Fluorescence emission in the liver PCF is gradually weakened and eventually diminished after 1 hour following ICG injection, while the gall bladder portion is gradually highlighted, indicating the discretion of ICG by bile to the gall bladder. At two hours post injection, the highlighted fluorescence emission moves from gallbladder to the middle and then the lower abdominal areas, indicating further transport of ICG through intestines. After several hours post injection of ICG, no fluorescence emission can be detected and projected.

**Fig 13:**
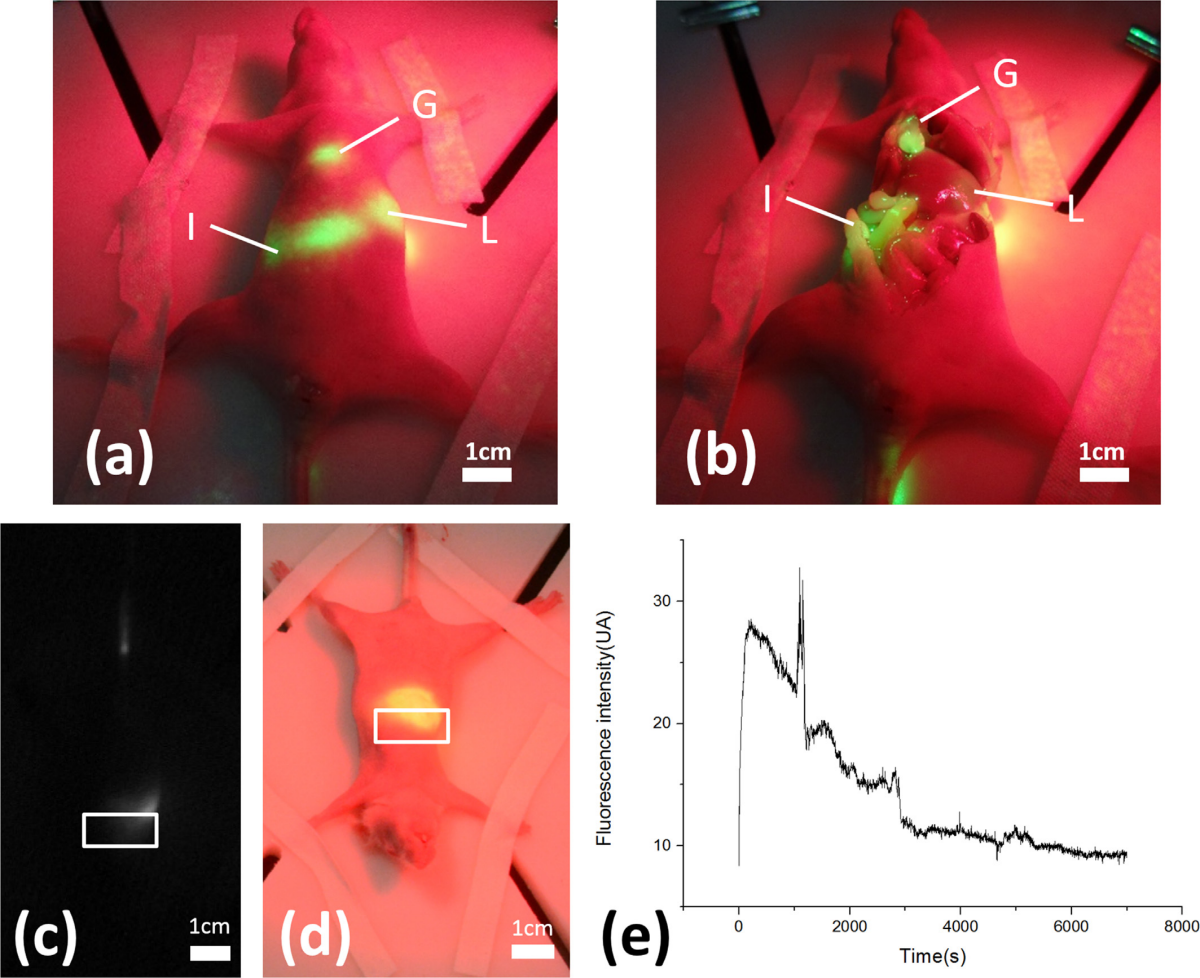
In vivo, ICG concentration–time course in mouse liver following ICG injection. (a) The nude mouse before dissection at 3 hours after ICG tail intravenous injection; (b) After the mouse is sacrificed by cervical vertebra dislocation, we dissect its abdomen and observe the anatomy map. (c) The fluorescence image captured by NIR camera. The area into the white frame is used to calculate fluorescence intensity. (d)The image of nude mouse under projective navigation system captured by nude eye. (e) Corresponding fluorescence intensity basis time courses.

Three hours after i.v. injection of the ICG solution, the animal is euthanized by cervical dislocation and then laparotomized for direct visualization of the ICG distribution in the peritoneal cavity. According to Fig 13A and 13B, a significant amount of ICG is accumulated in gallbladder, intestines and a small fraction of liver, while little ICG can be detected in skin. As we define a small region of interest (ROI) in the designated area of liver and calculate the averaged fluorescence intensity within the ROI over time, we are able to estimate the pharmacokinetic profile of ICG in liver after i.v. injection, as shown Fig 13C–13E. According to the figure, ICG distribution in liver follows a pattern of exponential decrease. A spike observed at the 16th minute post i.v. injection is induced by a sudden motion of the animal and does not represent the actual change of ICG concentration.

ICG is an FDA approved fluorescence agent that has been commonly used for clinical angiography. After i.v. injection, ICG is circulated, eliminated by liver, excreted in unmetabolized form into the bile, accumulated in the intestines through the bile duct, and eventually excreted with the feces [28, 34, 35]. Our in vivo imaging results are coincident with this classic model of ICG hepatic metabolism and in good agreement with previously published results [36–39]. This indicate that the projective navigation system can be used for in vivo fluorescence imaging of ICG distribution.

## Discussion

NIR fluorescence-guided surgery requires real-time acquisition and display of fluorescence images in a natural mode of visual perception. In this study, we develop a fluorescence navigation system for direct projection of disease margins on tissue surface. The technique successfully overcomes the limitations of the present NIR fluorescence imaging systems and free surgeons from visual distractions during a surgical procedure. With the current hardware and software configurations, our projective surgical navigation system is able to achieve an overall imaging speed higher than 60 frames per second, with a latency of 330 ms, a spatial sensitivity better than 0.5 mm in both vertical and horizontal directions, and a projective bias less than 1 mm after appropriate height correction. Comparison between the fluorescence images acquired by our system and by a commercial IVIS Lumina III small animal imaging system (Perkin Elmer, Waltham, MA) shows similar imaging sensitivity and dynamic range. And we compare the projective system with several other NIRF imaging system[18–20, 40–44] in Table 1 to show the differences.

**Table 1:** Comparison of the projective system with other NIFR systems.

Imaging System	Excitation Light Source	Spatial Resolution	Working Distance	Field of View (cm^2^)	Background Color Image
**Fluobeam**	Laser 690nm 2.6mW/cm^2^	0.17mm	20cm	28.27–50.27	NO
**SPY**	Laser 806nm 35.7mW/cm^2^	NS	30cm	56	NO
**PDE**	LED 760nm power NS	NS	15-25cm	NS	NO
**FLARE**	LED 670nm 4mW/cm^2^	125 to 625 μm	45.72cm (18inch)	3.7–169.5	YES
	LED 760nm 14mW/cm^2^				
**George Themelis’s prototype**	Laser 750nm	68.21 μm	21cm	1.5–107	YES
**Projective Imaging System**	LED 690nm 4.37–6.57 mW/cm²	<0.5mm	35cm	70–90	NO

Application of the projective navigation system is limited by the lack of cancer-targeting fluorescent dyes since ICG cannot bind with tumors or cancer tissues specifically. If more disease-targeting fluorescent dyes were to be developed, the projective surgical navigation system could be used to improve clinical outcomes for cancer resection and provide localization information for many tissue malignancies. Possible applications include preoperative tumor localization, determining the resection margin status[4] and identifying otherwise missed tumor foci[5]. In addition to cancer resection surgery, this technique can also be used in other clinical fields such as real-time projection of tissue vasculature, blood flow, oxygenation, and skeletal structure in for the guided plastic surgery.

The imaging system presented in this paper is a research prototype to demonstrate the concept of a novel surgical navigation method. Further engineering optimization and clinical validation works are necessary before the system can be deployed for clinical use. Especially, we will improve the system performance further by optimizing the hardware design, the communication protocol, and the image registration algorithm. First, topographical measurements will be integrated into the system so that a 3D profile of the surgical field can be used to correct the projection bias in real time. Second, the data communication protocol and the imaging processing algorithm will be optimized and simplified for increased navigation speed and reduced motion artifacts. To further simplify the calibration procedure, we will save the corresponding image transformation matrices at different projector-surgical plane distances in advance so that the specific calibration factors can be loaded automatically based on the detected height information without additional calibration operation. Third, we will optimize the system design to improve its portability and clinical utility. For example, the excitation light source, the cameras, and the projection module will be integrated in a compact enclosure and placed in a medical trolley for mobility. Finally, the integrated system will be tested in a clinical trial for image-guided surgery.

## Supporting Information

S1 File Data of Fig 4 Comparison of fluorescence detection capability. The concentration of ICG aqueous solution are 0, 0.0001, 0.0005, 0.001, 0.002, 0.003, 0.004, 0.006, 0.008, 0.01, 0.02, 0.03, 0.08, 0.1 mg/ml respectively. Fluorescence intensity is detected at each concentration of ICG aqueous solution by IVIS Lumina III and projective surgical navigation system. Average value and standard deviation of fluorescence intensity are calculated at the same time.
(XLSX)

S2 File Data of Fig 9 Phantom study to show the projective accuracy as the function of the height differences. The projection bias is detected with the height difference with and without height correction algorithm. Average value and standard deviation of projection bias are calculated at the same time.
(XLSX)

S3 File Data of Fig 13(E) Corresponding fluorescence intensity basis time courses. (XLSX)

## References

[1] Apantaku LM. Breast-conserving surgery for breast cancer. American Family Physician. 2002;66(12):2271–8. 12507165

[2] Weber WP, Engelberger S, Viehl CT, Zanetti-Dallenbach R, Kuster S, Dirnhofer S, et al Accuracy of frozen section analysis versus specimen radiography during breast-conserving surgery for nonpalpable lesions. World Journal of Surgery. 2008;32(12):2599–606. 10.1007/s00268-008-9757-8 18836763

[3] Smitt MC, Nowels K, Carlson RW, Jeffrey SS. Predictors of reexcision findings and recurrence after breast conservation. Int J Radiat Oncol Biol Phys. 2003;57(4):979–85. 14575828 10.1016/s0360-3016(03)00740-5

[4] Horst KC, Smitt MC, Goffinet DR, Carlson RW. Predictors of local recurrence after breast-conservation therapy. Clinical breast cancer. 2005;5(6):425–38. 15748463 10.3816/cbc.2005.n.001

[5] Waljee JF, Hu ES, Newman LA, Alderman AK. Predictors of re-excision among women undergoing breast-conserving surgery for cancer. Annals of surgical oncology. 2008;15(5):1297–303. 10.1245/s10434-007-9777-x 18259820

[6] Park CC, Mitsumori M, Nixon A, Recht A, Connolly J, Gelman R, et al Outcome at 8 years after breast-conserving surgery and radiation therapy for invasive breast cancer: influence of margin status and systemic therapy on local recurrence. J Clin Oncol. 2000;18(8):1668–75. 10764427 10.1200/JCO.2000.18.8.1668

[7] Mery E, Jouve E, Guillermet S, Bourgognon M, Castells M, Golzio M, et al Intraoperative fluorescence imaging of peritoneal dissemination of ovarian carcinomas. A preclinical study. Gynecologic oncology. 2011;122(1):155–62. 10.1016/j.ygyno.2011.02.035 21463889

[8] Crane L, Themelis G, Arts H, Buddingh K, Brouwers A, Ntziachristos V, et al Intraoperative near-infrared fluorescence imaging for sentinel lymph node detection in vulvar cancer: first clinical results. Gynecologic oncology. 2011;120(2):291–5. 10.1016/j.ygyno.2010.10.009 21056907

[9] Kitai T, Inomoto T, Miwa M, Shikayama T. Fluorescence navigation with indocyanine green for detecting sentinel lymph nodes in breast cancer. Breast cancer. 2005;12(3):211–5. 16110291 10.2325/jbcs.12.211

[10] van der Poel HG, Buckle T, Brouwer OR, Olmos RAV, van Leeuwen FW. Intraoperative laparoscopic fluorescence guidance to the sentinel lymph node in prostate cancer patients: clinical proof of concept of an integrated functional imaging approach using a multimodal tracer. European urology. 2011;60(4):826–33. 10.1016/j.eururo.2011.03.024 21458154

[11] Morita Y, Sakaguchi T, Unno N, Shibasaki Y, Suzuki A, Fukumoto K, et al Detection of hepatocellular carcinomas with near-infrared fluorescence imaging using indocyanine green: its usefulness and limitation. International journal of clinical oncology. 2013;18(2):232–41. 10.1007/s10147-011-0367-3 22200990

[12] Tan I-C, Maus EA, Rasmussen JC, Marshall MV, Adams KE, Fife CE, et al Assessment of lymphatic contractile function after manual lymphatic drainage using near-infrared fluorescence imaging. Archives of physical medicine and rehabilitation. 2011;92(5):756–64. e1. 10.1016/j.apmr.2010.12.027 21530723 PMC3109491

[13] Yamamoto T, Yamamoto N, Doi K, Oshima A, Yoshimatsu H, Todokoro T, et al Indocyanine green–enhanced lymphography for upper extremity lymphedema: a novel severity staging system using dermal backflow patterns. Plastic and reconstructive surgery. 2011;128(4):941–7. 10.1097/PRS.0b013e3182268cd9 21681123

[14] Gurfinkel M, Thompson AB, Ralston W, Troy TL, Moore AL, Moore TA, et al Pharmacokinetics of ICG and HPPH‐car for the Detection of Normal and Tumor Tissue Using Fluorescence, Near—infrared Reflectance Imaging: A Case Study. Photochemistry and Photobiology. 2000;72(1):94–102. 10911733 10.1562/0031-8655(2000)072<0094:poiahc>2.0.co;2

[15] Corlu A, Choe R, Durduran T, Rosen MA, Schweiger M, Arridge SR, et al Three-dimensional in vivo fluorescence diffuse optical tomography of breast cancer in humans. Optics express. 2007;15(11):6696–716. 19546980 10.1364/oe.15.006696

[16] Gioux S, Choi HS, Frangioni JV. Image-guided surgery using invisible near-infrared light: fundamentals of clinical translation. Molecular imaging. 2010;9(5):237 20868625 PMC3105445

[17] Yaroslavsky AN, Patel R, Wirth D. Devices and methods for optical pathology Google Patents; 2012.

[18] Vogt P, Bauer E, Graves K. Novadaq Spy Intraoperative Imaging System—current status. The Thoracic and cardiovascular surgeon. 2003;51(1):49–51. 12587091 10.1055/s-2003-37276

[19] Troyan SL, Kianzad V, Gibbs-Strauss SL, Gioux S, Matsui A, Oketokoun R, et al The FLARE™ intraoperative near-infrared fluorescence imaging system: a first-in-human clinical trial in breast cancer sentinel lymph node mapping. Annals of surgical oncology. 2009;16(10):2943–52. 10.1245/s10434-009-0594-2 19582506 PMC2772055

[20] Lee BT, Hutteman M, Gioux S, Stockdale A, Lin SJ, Ngo LH, et al The FLARE™ intraoperative near-infrared fluorescence imaging system: a first-in-human clinical trial in perforator flap breast reconstruction. Plastic and reconstructive surgery. 2010;126(5):1472 10.1097/PRS.0b013e3181f059c7 21042103 PMC2974179

[21] Liu Y, Bauer AQ, Akers WJ, Sudlow G, Liang K, Shen D, et al Hands-free, wireless goggles for near-infrared fluorescence and real-time image-guided surgery. Surgery. 2011;149(5):689–98. 10.1016/j.surg.2011.02.007 21496565 PMC3079879

[22] Liu Y, Njuguna R, Matthews T, Akers WJ, Sudlow GP, Mondal S, et al Near-infrared fluorescence goggle system with complementary metal–oxide–semiconductor imaging sensor and see-through display. Journal of biomedical optics. 2013;18(10):101303–. 10.1117/1.JBO.18.10.101303 23728180 PMC3667841

[23] Shao P, Ding H, Wang J, Liu P, Ling Q, Chen J, et al Designing a wearable navigation system for image-guided cancer resection surgery. Annals of biomedical engineering. 2014;42(11):2228–37. 10.1007/s10439-014-1062-0 24980159 PMC4332818

[24] Wu J-R, Wang M-L, Liu K-C, Hu M-H, Lee P-Y. Real-time advanced spinal surgery via visible patient model and augmented reality system. Computer methods and programs in biomedicine. 2014;113(3):869–81. 10.1016/j.cmpb.2013.12.021 24461259

[25] Fujiwara M, Mizukami T, Suzuki A, Fukamizu H. Sentinel lymph node detection in skin cancer patients using real-time fluorescence navigation with indocyanine green: preliminary experience. Journal of Plastic, Reconstructive & Aesthetic Surgery. 2009;62(10):e373–e8. 10.1016/j.bjps.2007.12.074 18556255

[26] Yuasa Y, Seike J, Yoshida T, Takechi H, Yamai H, Yamamoto Y, et al Sentinel lymph node biopsy using intraoperative indocyanine green fluorescence imaging navigated with preoperative CT lymphography for superficial esophageal cancer. Annals of surgical oncology. 2012;19(2):486–93. 10.1245/s10434-011-1922-x 21792510

[27] Miyashiro I, Miyoshi N, Hiratsuka M, Kishi K, Yamada T, Ohue M, et al Detection of sentinel node in gastric cancer surgery by indocyanine green fluorescence imaging: comparison with infrared imaging. Annals of surgical oncology. 2008;15(6):1640–3. 10.1245/s10434-008-9872-7 18379850

[28] Desmettre T, Devoisselle J, Mordon S. Fluorescence properties and metabolic features of indocyanine green (ICG) as related to angiography. Survey of ophthalmology. 2000;45(1):15–27. 10946079 10.1016/s0039-6257(00)00123-5

[29] Kang Y, Choi M, Lee J, Koh GY, Kwon K, Choi C. Quantitative analysis of peripheral tissue perfusion using spatiotemporal molecular dynamics. PLoS One. 2009;4(1):e4275 10.1371/journal.pone.0004275 19169354 PMC2626246

[30] Meier R, Thürmel K, Moog P, Noël PB, Ahari C, Sievert M, et al Detection of synovitis in the hands of patients with rheumatologic disorders: diagnostic performance of optical imaging in comparison with magnetic resonance imaging. Arthritis & Rheumatism. 2012;64(8):2489–98. 22421978 10.1002/art.34467

[31] Tanaka E, Choi HS, Humblet V, Ohnishi S, Laurence RG, Frangioni JV. Real-time intraoperative assessment of the extrahepatic bile ducts in rats and pigs using invisible near-infrared fluorescent light. Surgery. 2008;144(1):39–48. 10.1016/j.surg.2008.03.017 18571583 PMC2453786

[32] Michalet X, Weiss S. Using photon statistics to boost microscopy resolution. Proceedings of the National Academy of Sciences of the United States of America. 2006;103(13):4797–8. 16549771 10.1073/pnas.0600808103 PMC1458746

[33] Kaneko H, Horie J. Breathing movements of the chest and abdominal wall in healthy subjects. Respiratory care. 2012;57(9):1442–51. 10.4187/respcare.01655 22348414

[34] Yaseen MA, Yu J, Jung B, Wong MS, Anvari B. Biodistribution of encapsulated indocyanine green in healthy mice. Molecular pharmaceutics. 2009;6(5):1321–32. 10.1021/mp800270t 19799463 PMC2758533

[35] Cherrick GR, Stein SW, Leevy CM, Davidson CS. Indocyanine green: observations on its physical properties, plasma decay, and hepatic extraction. Journal of Clinical Investigation. 1960;39(4):592. 13809697 10.1172/JCI104072 PMC293343

[36] El‐Desoky A, Seifalian A, Cope M, Delpy D, Davidson B. Experimental study of liver dysfunction evaluated by direct indocyanine green clearance using near infrared spectroscopy. British journal of surgery. 1999;86(8):1005–11. 10460634 10.1046/j.1365-2168.1999.01186.x

[37] Hillman EM, Moore A. All-optical anatomical co-registration for molecular imaging of small animals using dynamic contrast. Nature photonics. 2007;1(9):526–30. 18974848 10.1038/nphoton.2007.146 PMC2575379

[38] Hillman EM, Amoozegar CB, Wang T, McCaslin AF, Bouchard MB, Mansfield J, et al In vivo optical imaging and dynamic contrast methods for biomedical research. Philosophical Transactions of the Royal Society of London A: Mathematical, Physical and Engineering Sciences. 2011;369(1955):4620–43. 10.1098/rsta.2011.0264 PMC3263788 22006910

[39] Saxena V, Sadoqi M, Shao J. Polymeric nanoparticulate delivery system for Indocyanine green: biodistribution in healthy mice. International journal of pharmaceutics. 2006;308(1):200–4. 16386861 10.1016/j.ijpharm.2005.11.003

[40] Themelis G, Yoo JS, Soh K-S, Schulz R, Ntziachristos V. Real-time intraoperative fluorescence imaging system using light-absorption correction. Journal of biomedical optics. 2009;14(6):064012–9. 10.1117/1.3259362 20059250

[41] Hutteman M, Mieog J, Van der Vorst J, Dijkstra J, Kuppen P, van der Laan A, et al Intraoperative near-infrared fluorescence imaging of colorectal metastases targeting integrin α v β 3 expression in a syngeneic rat model. European Journal of Surgical Oncology (EJSO). 2011;37(3):252–7. 21215590 10.1016/j.ejso.2010.12.014

[42] Mieog JSD, Vahrmeijer AL, Hutteman M, Van der Vorst JR, Van Hooff MD, Dijkstra J, et al Novel intraoperative near-infrared fluorescence camera system for optical image-guided cancer surgery. Molecular imaging. 2010;9(4):223 20643025

[43] Schaafsma BE, Mieog JSD, Hutteman M, Van der Vorst JR, Kuppen PJ, Löwik CW, et al The clinical use of indocyanine green as a near‐infrared fluorescent contrast agent for image‐guided oncologic surgery. Journal of surgical oncology. 2011;104(3):323–32. 10.1002/jso.21943 21495033 PMC3144993

[44] Tagaya N, Yamazaki R, Nakagawa A, Abe A, Hamada K, Kubota K, et al Intraoperative identification of sentinel lymph nodes by near-infrared fluorescence imaging in patients with breast cancer. The American Journal of Surgery. 2008;195(6):850–3. 10.1016/j.amjsurg.2007.02.032 18353274

